# The importance of examining movements within the US health care system: sequential logit modeling

**DOI:** 10.1186/1472-6963-10-269

**Published:** 2010-09-10

**Authors:** Chioun Lee, Stephanie L Ayers, Jennie Jacobs Kronenfeld, Jemima A Frimpong, Patrick A Rivers, Sam S Kim

**Affiliations:** 1 Department of Sociology, Rutgers University, 26 Nichol Avenue, New Brunswick, NJ 08901, USA; 2 Southwest Interdisciplinary Research Center, Arizona State University, 411 N. Central Ave, Suite 720, Phoenix, AZ, USA; 3 Sociology Program, School of Social and Family Dynamics, Arizona State University, P.O. Box 873701, Tempe, AZ 85287, USA; 4 Mailman School of Public Health, Columbia University, 60 Haven Avenue, Suite B-2, New York, NY 10032, USA; 5 Health Care Management Program, School of Allied Health, Southern Illinois University, 1365 Douglas Drive, Carbondale, IL 62901, USA; 6 Dartmouth Institute for Health Policy & Clinical Practice, Center for Education, Dartmouth College, 30 Lafayette Street, Lebanon, NH 03766, USA

## Abstract

**Background:**

Utilization of specialty care may not be a discrete, isolated behavior but rather, a behavior of sequential movements within the health care system. Although patients may often visit their primary care physician and receive a referral before utilizing specialty care, prior studies have underestimated the importance of accounting for these sequential movements.

**Methods:**

The sample included 6,772 adults aged 18 years and older who participated in the 2001 Survey on Disparities in Quality of Care, sponsored by the Commonwealth Fund. A sequential logit model was used to account for movement in all stages of utilization: use of any health services (i.e., first stage), having a perceived need for specialty care (i.e., second stage), and utilization of specialty care (i.e., third stage). In the sequential logit model, all stages are nested within the previous stage.

**Results:**

Gender, race/ethnicity, education and poor health had significant explanatory effects with regard to use of any health services and having a perceived need for specialty care, however racial/ethnic, gender, and educational disparities were not present in utilization of specialty care. After controlling for use of any health services and having a perceived need for specialty care, inability to pay for specialty care via income (AOR = 1.334, CI = 1.10 to 1.62) or health insurance (unstable insurance: AOR = 0.26, CI = 0.14 to 0.48; no insurance: AOR = 0.12, CI = 0.07 to 0.20) were significant barriers to utilization of specialty care.

**Conclusions:**

Use of a sequential logit model to examine utilization of specialty care resulted in a detailed representation of utilization behaviors and patient characteristics that impact these behaviors at all stages within the health care system. After controlling for sequential movements within the health care system, the biggest barrier to utilizing specialty care is the inability to pay, while racial, gender, and educational disparities diminish to non-significance. Findings from this study represent how Americans use the health care system and more precisely reveals the disparities and inequalities in the U.S. health care system.

## Background

The Institute of Medicine [1] suggests that lack of access to specialty care results in hospitalization and long-term negative effects on patient health outcomes. Variations in the use of specialty care and barriers to accessing specialty care are attributed to patient factors (e.g., age, gender, race/ethnicity, insurance status) and provider factors (e.g., referral patterns) [2-8]. In order to better understand disparities in health care utilization, it is imperative to investigate how both patient factors and provider factors impact variations in specialty care. These variations are important factors accounting for disparities in health care utilization and health outcomes in the U.S.

Patient factors that predict use of specialty care (e.g. income, education, gender, race/ethnicity, health insurance status, and self-rated health [5-7,9,10]) influence perceptions and need for utilization [8,11-13], create barriers that decrease the probability of utilizing specialty care [14], and lead to negative health care experiences [15]. Utilization of specialty care is lower for females, racial/ethnic minorities, and individuals who lack private health insurance, and those in good health [2,3,6-8,12,14,16-19]. Patients without private health insurance show decreased utilization of specialists when compared to privately insured patients [2,3,20-22]. Studies also show that income and education impact utilization of specialty care by influencing knowledge and understanding for the need of specialized care [8,11-13]. In addition to disparities in specialty care utilization, patients who are uninsured, underinsured, or economically disadvantaged also experience barriers to accessing specialty care services (e.g., lack of physician referrals) [4,5,23] most often because specialty care involves expensive treatments and procedures that require extensive resources.

Provider factors may also serve as barriers to patients' access to specialists. In the U.S. health care system primary care physicians have two distinct tasks: providing first contact care and coordinating delivery and referrals to other health services [22,24]. Because of this, primary care physicians have a unique role in specialty care utilization [24,25]. For example, "administrative mechanisms such as preauthorization (gatekeeping) may decrease specialty visits" [15].

Previous studies have examined specialty care utilization without examining the organization of the U.S. health care system [2,11,26,27]. Studies have overlooked a possible determinant of specialty care utilization: it is not an isolated and discrete behavior, but rather, may be a behavior that is part of a sequential movement within the U.S. health care system. Specifically, in many health care organizations, patients must first obtain a referral in order to gain access to a specialist [28]. By examining specific sequential movements within the health care system, our study applies the most current Andersen Behavioral Model of Health Service Use which takes into account the accessibility and structural components within health care organizations. Thus, the aim of our research is to analyze utilization of specialty care using the modified Andersen Behavioral Model of Health Service Use [27]. The Behavioral Model illustrates connections between predisposing, enabling, and need characteristics to predict use of health services. Each component makes independent and compounding contributions to predicting utilization of health services [27]. This study employs a sequential logit model to account for the effects of predisposing, enabling and need factors in all stages of utilization of health services. In this study, we explore two questions: 1) What are the differences in the determinants of health care utilization when considering sequential movements of health seeking behaviors? and 2) How does the Andersen Behavioral Model of Health Service Use explain utilization of specialty care after controlling for utilization of general health care services and perceiving a need for specialty care?

## Methods

### Data

Data for this study come from the 2001 Survey on Disparities in Quality of Health Care, publically available at http://www.cmwf.org/surveys/surveys.htm. The data were collected using a random-digital-dialing telephone survey of U.S. adults aged 18 years and older with African-Americans, Hispanics, and Asians oversampled. The overall response rate for this survey is 54.3% (contacted rate: 75.2%, cooperated rate: 72.8% and completed rate 99.1%). Missing data ranged from 0.6% to 18% with the majority of missing data coming from non-responses to annual income. In order to reduce loss of information and account for missing data, we applied the multiple imputation procedure using STATA 9.0 [29], which resulted in a starting sample size of 6,722.

### Dependent Variables

**Figure 1:**
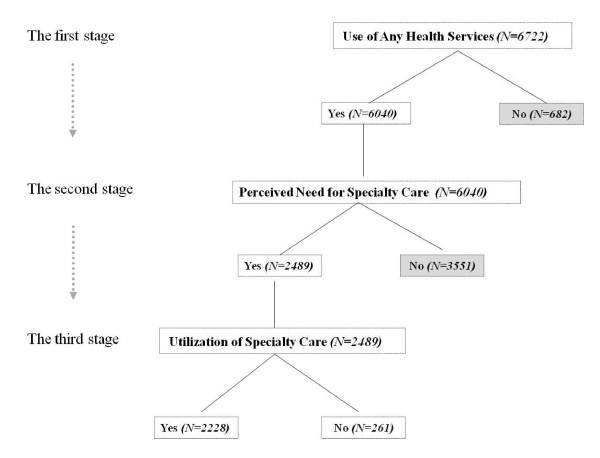
**Sequential Logit Model**.

Because utilization of specialty care is not a discrete, isolated behavior but rather, may be a behavior of sequential movements within the health care system, the dependent variables used in this study represent three stages of sequential health utilization behaviors (Figure 1). These three stages are nested within the previous stage. Thus, individuals must pass through the first stage in order to be included in the second stage, and similarly, individuals must pass through the second stage in order to be included in the third stage.

The first stage, *use of any health services*, was measured by the question, "Have you visited a doctor or medical clinic for any reason, including check-ups or visits to the emergency room or hospital, in the last two years?" This variable was coded as no health service visit (i.e., 0) and yes, had a health service visit in the past two years (i.e., 1). The second stage, *perceived need for specialty care*, by the physician or patient, only included respondents who used any health services in the past two years and was measured by the question, "In the past two years, was there any time when you or your doctor thought you needed to see a specialist?" This variable was coded as no perceived need for specialty care (i.e., 0) and yes, had a perceived need for specialty care (i.e., 1). The third stage, *utilization of specialty care*, only included respondents who had a perceived need for specialty care and was measured by the question, "Were you able to see a specialist?" This variable was coded as no specialty care visit (i.e., 0) and yes, had a specialty care visit (i.e., 1).

### Independent Variables

Three sets of explanatory variables account for variations in the sequential logit model. Before operationalization of any explanatory variables occurred, non-linear relationships between the three dependent variables and independent variables (e.g, age, health status, and chronic conditions) were first confirmed. The first set of these explanatory variables measures the predisposing characteristics of gender, age, race/ethnicity, and education. Males were the reference group for gender, while age was a continuous variable ranging from 18 to 97. Race/ethnicity included categories for non-Hispanic black, Hispanic, and other racial groups with Non-Hispanic white as the reference group. Education, a continuous variable, ranged from 1 (i.e., none or primary grade level) to 6 (i.e., beyond a Master's degree).

The second set of explanatory variables measured the enabling resources of income and insurance status. Income, a continuous variable, ranged from 1 (i.e., < $25,000) to 5 (i.e., ≥ $75,000). Insurance continuity measured respondents' status of health insurance over the previous 12-month period and was coded into three categories: continuous insurance, no insurance, and unstable insurance (i.e., having health insurance to being uninsured, or being uninsured to having health insurance). Continuous insurance was the reference group. Type of health insurance (i.e., private health insurance, Medicaid, Medicare, and no health insurance) was included in previous analyses, however, there were no significant differences between private insurance, Medicaid, and Medicare in having a perceived need for specialty care and utilization of specialty care after adjusting covariates, while insurance continuity significantly accounted for variation of utilizing any health services.

The last set of explanatory variables accounted for need factors and included health status and number of chronic diseases. Health status ranged from 1 (i.e., excellent) to 5 (i.e., poor) and was coded as such in order to maintain a consistent direction with higher numbers representing poorer health. Number of chronic diseases ranged from 0 (i.e., no chronic disease) to 4 (i.e., 4 or more chronic diseases).

### Statistical Analysis

We modeled the effects of sequential movements within the health care system in three stages: (1) use of any health services, (2) perceived need for specialty care, and (3) utilization of specialty care (Figure 1). A sequential logit model is an appropriate method to analyze the way these sequential decisions are made [30]. The sequential logit model assumes that individuals make choices, but often these choices are not made simultaneously. Rather, individuals make a number of sub-choices based on previous choices, [31] thus "the response categories [are] perceived as a sequence with stages. The response in a later stage is nested in the response in an earlier stage" [[32], p.25].

The decision-making tree, as shown in Figure 1, consists of three sets of binary choices [32]:

y_1 _= 0   if the individual does not utilize any health services (N = 682)

y_1 _= 1   if the individual utilizes any health services (N = 6,040)

y_2 _= 0   if the individual does not have a perceived need for specialty care (N = 3,551)

y_2 _= 1   if the individual has a perceived need for specialty care (N = 2,489)

y_3 _= 0   if the individual does not utilize specialty care (N = 261)

y_3 _= 1   if the individual utilizes specialty care (N = 2,228)

Analytic weights were used in all sequential logit models to adjust for the complex sampling design and to represent the U.S. population. STATA 9.0 [29] was used for the analyses because of its ability to run analyses with complex sampling designs, such as weight, cluster, and strata variables, so that estimates and standard errors are unbiased. The Institution Review Board at Arizona State University granted an exemption to this study (Protocol Number: 0704001814).

## Results

Descriptive statistics are shown in Table 1. Results for the dependent variables show that 89% of respondents used any health services within the past two years, 42% had perceived a need for specialty care after using any health services, and 89% utilized specialty care after having a perceived need for specialty care. Fewer Hispanics, non-Hispanic Blacks, and individuals with no health insurance utilized specialty care compared to using any health services and having a perceived need for specialty care. Conversely, more non-Hispanic whites utilized specialty care when compared to using any health services and having a perceived need for specialty care. This pattern is also similar for older individuals, individuals with stable health insurance and those with higher education, income, and number of chronic diseases.

**Table 1:** Descriptive Statistics for Variables Used in the Analyses

	The first stage:	The second stage:	The third stage:	Range	
	
	Use of Any Health Services	Perceived Need for Specialty Care	Utilization of Specialty Care		
	(0 = no, 1 = yes) (N = 6,722)	(0 = no, 1 = yes) (n = 6,040)	(0 = no, 1 = yes) (n = 2,489)		
Variables	**Mean (SE^1^)**	**Mean (SE^1^)**	**Mean (SE^1^)**	**Min**	**Max**

**Dependent variables**					
Use of Any Health Services	0.89 (0.006)			0	1
Perceived Need for Specialty Care		0.42 (0.009)		0	1
Utilization of Specialty Care			0.89 (0.009)	0	1
**Independent variables**					
***Predisposing Characteristics***					
Female	0.54 (0.009)	0.57 (0.010)	0.59 (0.015)	0	1
Age	45.29 (0.307)	45.75 (0.327)	48.37 (0.499)	18	97
Race/Ethnicity					
Non-Hispanic White	0.70 (0.007)	0.71 (0.007)	0.73 (0.011)		
Non-Hispanic Black	0.11 (0.004)	0.11 (0.005)	0.09 (0.006)	0	1
Hispanic	0.11 (0.004)	0.10 (0.004)	0.08 (0.006)		
Other	0.09 (0.005)	0.08 (0.005)	0.10 (0.008)		
Education	3.71 (0.022)	3.74 (0.023)	3.84 (0.036)	1	6
***Enabling resources***					
Income	2.90 (0.024)	2.91 (0.025)	2.95 (0.040)	1	5
Insurance continuity					
Stable health insurance	0.78 (0.007)	0.79 (0.007)	0.81 (0.011)		
Unstable health insurance	0.07 (0.005)	0.08 (0.005)	0.08 (0.008)	0	1
No health insurance	0.15 (0.006)	0.13 (0.006)	0.11 (0.009)		
***Need***					
Poor health status	2.46 (0.019)	2.49 (0.021)	2.76 (0.033)	1	5
Number of chronic diseases	0.75 (0.018)	0.82 (0.020)	1.14 (0.034)	0	4

^1. ^SE=Standard Error

Results from the sequential logit models for the three stages of health service utilization are shown in Table 2. Because all dependent variables were assigned binary coding, results are presented in adjusted odds ratios (AOR) with 95% confidence intervals (95% CI).

**Table 2:** Sequential Logistic Regression Models for Dependent Variables at Each Stage.

	The first stage:		The second stage:		The third stage:	
	Use of Any Health Services		Perceived Need for Specialty are		Utilization of Specialty C are	
	(N = 6,722)		(N = 6,040)		(n = 2,489)	
	**Odds ratio**	**95% CI**	**Odds ratio**	**95% CI**	**Odds ratio**	**95% CI**

**PREDISPOSING CHARACTERISTICS**						
Female	2 83 ***	2.20 - 3.65	1.19 *	1.01 - 1.40	0.97	0.62 - 1.52
Age	0.99	0.99 - 1.01	1.01	0.99 - 1.01	1.03 ***	1.01 -1.04
Race^1^						
Non-Hispanic Black	1.02	0.71 - 1.45	0.71 **	0.57 - 0.89	1.85	0.99 -3.47
Hispanic	0.71 *	0.51 - 0.98	0.82	0.64 - 1.05	1.12	0.64 -1.96
Other	0.64 *	0.44 - 0.92	1.17	0.89 - 1.53	0.76	0.37 -1.57
Education	1.31 ***	1.17 - 1.46	1.24 ***	1.15 - 1.34	0.92	0.74 -1.14
**ENABLING RESOURCES**						
Income	1.03	0.91 - 1.17	1.08 *	1.01 - 1.17	1.33 **	1.09 -1.62
Insurance continuity^2^						
Unstable health insurance	1.23	0.73 - 2.08	1.25	0.92 - 1.70	0.26 ***	0.14 -0.48
No health insurance	0 45 ***	0.33 - 0.62	0.91	0.70 - 1.17	0.12 ***	0.07 -0.20
**NEED**						
Poor health status	1.22 * *	1.07 - 1.40	1.42 ***	1.30 - 1.56	0.79 *	0.64 -0.99
Number of chronic diseases	2.53 ***	1.91 - 3.37	1.53 ***	1.39 - 1.68	0.96	0.76 -1.21

*p < .05 ** p < .01 ***p < .001

Reference Categories: Non-Hispanic White; Stable Insurance

### Use of Any Health Services

The predisposing characteristics of gender, race/ethnicity, and education had significant effects on utilization of any health services. Females were nearly three times more likely to use any health services when compared to males (AOR = 2.83, 95% CI: 2.20, 3.65). Hispanics and other racial/ethnic groups were less likely to use any health services when compared to non-Hispanic whites (AOR = 0.71, 95% CI: 0.51, 0.98; and AOR = 0.64, 95% CI: 0.44, 0.92, respectively); however, there was no significant difference between non-Hispanic whites and non-Hispanic blacks (AOR = 1.02, 95% CI: 0.71, 1.45). As expected, education was positively associated with using any health services (AOR = 1.31, 95% CI: 1.17, 1.46).

Insurance status was the only enabling resource that had a significant influence on predicting use of any health services. Those without health insurance during the last 12 months were less likely to use any health services when compared to those with stable health insurance, regardless of health status (AOR = 0.45, 95% CI: 0.33, 0.62). The need variables, health status and number of chronic diseases, also had significant effects on using any health services. Respondents with a poorer health status and those with more chronic diseases were more likely to use any health services (AOR = 1.22, 95% CI: 1.07, 1.40; and AOR = 2.53, 95% CI: 1.91, 3.37, respectively).

### Perceived Need for Specialty Care

The predisposing characteristics of gender, race/ethnicity, and education had significant influences on predicting having a perceived need for specialty care. After using any health services, females were more likely to have a perceived need for specialty care when compared to males (AOR = 1.19, 95% CI: 1.01, 1.40). Non-Hispanic blacks had significantly lower rates of having a perceived need for specialty care when compared to non-Hispanic whites (AOR = 0.71, 95% CI: 0.57, 0.89). Education was positively associated with having a perceived need for specialty care (AOR = 1.24, 95% CI: 1.15, 1.34).

Income, an enabling resource, had positive effects on having a perceived need for specialty care (AOR = 1.08, 95% CI: 1.01, 1.17), whereas, insurance status was not significant. As expected, need variables significantly impacted having a perceived need for specialty care. Individuals with a poorer health status and more chronic diseases were more likely to have a perceived need for specialty care (AOR = 1.42, 95% CI: 1.30, 1.56 and AOR = 1.53, 95% CI: 1.39, 1.68, respectively).

### Utilization of specialty care

Age and race/ethnicity were the predisposing characteristics that were significant predictors of utilizing specialty care. Older respondents were more likely to use specialty care after perceiving a need for such care (AOR = 1.03, 95% CI: 1.01, 1.04). The enabling resources, income and insurance continuity, had the strongest effect on using specialty care when compared to use of any health care. For example, every one unit increase of family income increased utilization of specialty care by 33% (AOR = 1.33, 95% CI: 1.09, 1.62). Insurance continuity also had a remarkable effect on use of specialty care. Those with unstable or no insurance were significantly less likely to utilize specialty care when compared to those with stable insurance (AOR = 0.26, 95% CI: 0.14, 0.48; and AOR = 0.12, 95% CI: 0.07, 0.20, respectively). When compared to the dependent variables use of any health services and perceived need for specialty care, the effect of insurance continuity was evident; however, due to the difference in sample sizes, no further comparisons were possible. Health status was the only need factor significantly related to utilizing specialty care. Interestingly, respondents who reported a poorer health status had a lower likelihood of utilizing specialty care (AOR = 0.79, 95% CI: 0.64, 0.99).

## Discussion

This study sought to examine utilization of specialty care while controlling for sequential movements within the health care system, an approach often underestimated in prior studies [2,3,6,11,18,26,27]. Employing the Andersen Behavioral Model of Health Service Use [25], our study built upon the 2005 expanded model and broaden the applicability of this model to understanding and explaining movements within the health care system. Our study demonstrates the importance of controlling for perception of need for specialty care. We find that enabling resources, not predisposing characteristics, are the main barriers to utilizing specialty care. Enabling resources have the greatest effect on utilization of specialty care when controlling for perception of need of specialty care and any use of health services. Inability to pay for specialty care, due to low income or health insurance, is the most significant barrier to utilization of specialty care.

Numerous studies that have focused on the relationship between sociodemographic variables and utilization of specialty care have not accounted for whether there is a perceived a need for specialty care [2,3,6,11,14,26,27]. These studies overlook the fact that utilization of specialty care may not be an isolated or discrete behavior, but a behavior that requires sequential movements within the health care system. Previous studies show that if the issue of perception of need for specialty care is not considered, different factors may have an impact on the use of a specialist [2,6,7,11,14,26,27]. When perception of need for specialty care is not controlled for, the predisposing characteristics and need variables are significant. For example, studies show that gender, educational, racial and health inequalities are important in utilizing specialists [2,6,7,11], while enabling factors such as income and insurance do not impact specialty care utilization [7,11,14,26].

This study adds to the body of literature on utilization of specialty care and provides a statistical methodology to examine the relationship between sociodemographic variables and utilization of specialty care while accounting for the issue of perceived need for specialty care. Our study demonstrates the importance of controlling for perception of need for specialty care, resulting in enabling characteristics being the main barriers to utilizing specialty care. Enabling resources have the greatest effect on utilization of specialty care when controlling for perception of need of specialty care and any use of health services. Inability to pay for specialty care, due to low income or lack of health insurance, is the most significant barrier to utilization of specialty care. Contrary to previous studies, insurance continuity did not impact the odds of perceiving a need for specialty care [2,23]. Individuals with unstable or no insurance had dramatically lowered odds of using specialty care after perceiving a need for specialty care. Likewise, income did not have a strong association with perception of need for specialty care as assessed by either the physician or the patient, but did impact the likelihood of seeing a specialist. These findings indicate that enabling resources present barriers to utilizing specialty care even when people perceive the need for specialty care. Those without the ability to pay, regardless of race/ethnicity, age or gender, did not utilize a specialist.

Our research reveals the stage in the utilization process where predisposing factors and need factors matter. Predisposing factors (e.g., gender, race/ethnicity, and education) and need factors (e.g., poor health status and chronic diseases) are not significant predictors of specialty care utilization once perception of need for specialty care is controlled. However, predisposing factors and need factors do significantly impact utilization of any health services and having a perceived need for specialty care. Although they do not directly impact specialty care utilization, predisposing factors and need factors act as important gatekeepers for those individuals who receive referrals. In the context of prior research, our finding that race/ethnicity is not a significant predictor of utilization of specialty care was unexpected. Because minority populations have poorer health status and are less likely to be insured [33], we expected these mechanisms to translate to racial differences in use of specialty care. Our findings suggest that there may be other enabling factors that are systematically excluded in analysis on racial variations in health and health care.

There are, however, several limitations to this study. There was no specific question regarding use of primary care. The dependent variable "any use of health services" also contains utilization of specialty care; however, our statistical methodology reduces this limitation because it accounts for conditional probabilities on the appropriate subsamples. In order to accurately represent the regressive movements for utilization of health services, future research will begin the sequential logit model with utilization of primary care instead of general use of health services. In addition, a more suitable question on referral for specialty care would have been one that asked whether a referral was received as well as the exact type of health insurance the individual is receiving, since that may also impact referral to specialty care. The question asked in this study limited the discussion to having a perceived need for specialty care, either by the physician or patient, rather than receiving a referral for specialty care. Second, the data were cross-sectional in nature and may not be the best estimate of the cumulative effects of predisposing, enabling, and need factors on movements of health service utilization. Future research should include longitudinal data in order to better understand the cumulative effects over time as individuals move through the health care system. Third, 18% of the respondents did not report income. Cross tabulations indicate that respondents who did not report income exhibited a normal distribution with respect to education (i.e., those with low and high education were more likely to report income than those with approximately two years of college). Although this study used imputed data with the results not differing greatly, the effects for income might be biased. Fourth, although extensive efforts were used to obtain a generalizable sample, the data were collected through random digit dialing. It is necessary to point out that certain populations, particularly low socioeconomic households, may systematically excluded in the sample because the household lacked a phone. However, in prior studies the bias this can produce is small [34]. Fifth, although not addressed in our study, perceptions of need for specialty referral might differ based on health literacy. Studies have shown that health literacy can impact understanding appointment cards [35], successful navigation of the health care system [36], and overall health outcomes [37]. Future research should investigate the role health literacy play in the sequential movements within the health care system.

## Conclusions

Based on these findings, there is a need for further studies to determine factors that specifically facilitate access to and utilization of specialty care for populations who have inadequate enabling resources. Sequential movements within the health care system, especially utilization of specialty care provides a platform for future research. This study addressed several important issues, both at the individual level and systems level that impact the understanding of utilizing specialty care, therefore, increasing positive health outcomes for patients. It is critical to model research that accurately describes patients' movement through the health care system. Findings from this study's sequential logit model represent how Americans use the health care system, and more precisely reveals the disparities and inequalities in the U.S. health care system. After controlling for sequential movements through the health care system, racial and gender inequalities disappear, and the biggest barrier to utilizing specialty care is the inability to pay. Understanding the exact barriers to receiving specialty care may reduce hospitalizations and long-term negative health effects on patient outcomes. This finding may also contribute to the current U.S. debate about modifications in the health care delivery system. This study demonstrates the importance of inability to pay for health care, either through low income or lack of health insurance, as the most significant barrier to utilization of specialty care. With the recent passage of the Obama health insurance reforms, more people in the US should have health insurance in the future, and this may help to improve utilization of specialty care. The approach used in this paper can be used in future years, once those reforms have been implemented, to see whether they do impact use of specialty care.

## Competing interests

The authors declare that they have no competing interests.

## Authors' contributions

CL conceptualized the study, participated in the analysis of the data, and drafted the manuscript. SLA participated in the analysis of the data and drafted the manuscript. JJK conceptualized the study and drafted the manuscript. JAF drafted the manuscript. PAR conceptualized the project and drafted the manuscript. SSK drafted the manuscript. All authors read and approved the final manuscript

## Pre-publication history

The pre-publication history for this paper can be accessed here:

http://www.biomedcentral.com/1472-6963/10/269/prepub
